# LncRNA SH3PXD2A-AS1 facilitates cisplatin resistance in non-small cell lung cancer by regulating FOXM1 succinylation

**DOI:** 10.1186/s12885-024-12624-9

**Published:** 2024-07-17

**Authors:** Yunfeng Chen, Siyan Wu, Yu Han, Hai Shi, Jieqing Yuan, Wenjie Cui

**Affiliations:** 1 Cancer Institute, Xuzhou Medical University, No. 206, Tongshan Road, Xuzhou, Jiangsu 221116 China; 2 Department of Respiratory and Critical Care Medicine, The Affiliated Xuzhou Municipal Hospital of Xuzhou Medical University, No. 269, University Road, Tongshan District, Xuzhou, Jiangsu 221116 China

**Keywords:** Non-small cell lung cancer, SH3PXD2A-AS1, SIRT7, Succinylation, DDP resistance, FOXM1

## Abstract

**Background:**

Long noncoding RNAs (lncRNAs) play vital regulatory functions in non-small cell lung cancer (NSCLC). Cisplatin (DDP) resistance has significantly decreased the effectiveness of DDP-based chemotherapy in NSCLC patients. This study aimed to investigate the effects of SH3PXD2A antisense RNA 1 (SH3PXD2A-AS1) on DDP resistance in NSCLC.

**Methods:**

Proliferation and apoptosis of DDP-resistant NSCLC cells were detected using cell counting kit-8 and flow cytometry assays. The interaction between SH3PXD2A-AS1 and sirtuin 7 (SIRT7) was assessed using co-immunoprecipitation (Co-IP), RNA pull-down, RNA immunoprecipitation (RIP), RNA fluorescence in situ hybridization, and immunofluorescence assays, while succinylation (SUCC) of Forkhead Box M1 (FOXM1) was analyzed by IP and Western blot assays. The role of SH3PXD2A-AS1 in vivo was explored using a xenografted tumor model.

**Results:**

Expression of SH3PXD2A-AS1 was found elevated in DDP-resistant NSCLC cells, while it’s knocking down translated into suppression of cell viability and promotion of apoptosis. Moreover, silencing of SH3PXD2A-AS1 resulted in decreased FOXM1 protein level and enhanced FOXM1-SUCC protein level. The SIRT7 was found to interact with FOXM1, translating into inhibition of FOXM1 SUCC at the K259 site in human embryonic kidney (HEK)-293T cells. Overexpressing of SIRT7 reversed the increase of FOXM1-SUCC protein level and apoptosis, and the decrease of cell viability induced by silencing of SH3PXD2A-AS1. In tumor-bearing mice, SH3PXD2A-AS1 inhibition suppressed tumor growth and the protein levels of Ki67, SIRT7, and FOXM1.

**Conclusion:**

SH3PXD2A-AS1 promoted DDP resistance in NSCLC cells by regulating FOXM1 SUCC via SIRT7, offering a promising therapeutic approach for NSCLC.

**Supplementary Information:**

The online version contains supplementary material available at 10.1186/s12885-024-12624-9.

## Introduction

Lung cancer is a significant global health issue characterized by increasing incidence and mortality rates, with non-small cell lung cancer (NSCLC) accounting for 80–85% of cases [1, 2]. Multiple factors may contribute to the onset of lung cancer, including smoking, air pollution, radiation, and genetics, where most NSCLC patients are typically diagnosed in advanced stages, thus resulting in only 15% of the 5-year survival rate [3]. The NSCLC treatment options include radiotherapy, chemotherapy, antibody-drug conjugate, etc., [1], where most of the NSCLC are treated with cisplatin (DDP)-based chemotherapy. However, DDP resistance often develops during treatment, resulting in poor clinical outcomes for DDP-resistant patients [4]. Thus, it is imperative to explore effective treatment modalities for DDP-resistant NSCLC.

Long non-coding RNAs (lncRNAs) are a class of transcripts (> 200 nucleotides) with limited protein-coding ability [5], and play multiple roles in the cell nucleus and cytoplasm. The nucleus-located lncRNAs are involved in gene regulation at the epigenetic and transcriptional levels [6, 7], and cytoplasmic lncRNAs are involved in gene regulation at the post-transcriptional and translational levels [8, 9]. Mounting evidence suggests that lncRNAs play a role in the progression of multiple tumors. Moreover, previous research has demonstrated that SH3PXD2A antisense RNA 1 (SH3PXD2A-AS1) promotes the progression of various types of tumors [10–12], and was reported to be highly expressed in NSCLC and, hence, poor overall survival [2].

Succinylation (SUCC) is an essential post-translational modification, regulating protein functions and different signaling pathways [13]. Moreover, SUCC is also involved in various metabolic processes and disease progression. The succinylated proteins have been found to exert either promotive or inhibitive effects on the development of different cancers [14, 15]. Sirtuins (SIRTs) are class III histone deacetylases involved in multiple physiological and pathological processes [16], where SIRT1-7 in mammals have been found to regulate metabolism, cell cycle, lifespan, diseases, etc. [16, 17]. SIRT7, found exclusively in eukaryotes, is the least researched sirtuin, and has been linked to various non-neoplastic and tumorous diseases, such as cardiovascular diseases, obesity and NSCLC [16, 18, 19]. A prior study suggests that chromatin SUCC increases when SIRT7 is depleted, indicating a potential desuccinylation role for SIRT7 [20].

Forkhead box protein M1 (FOXM1) is a key proliferation-related transcription factor that is closely involved in cell proliferation, self-renewal, and tumorigenesis [21]. A recent study reports that AlkB homolog 5 was involved in promoting silica-induced pulmonary fibrosis by targeting FOXM1 [22]. Moreover, FOXM1 knockdown has been found to enhance NSCLC immunotherapy by suppressing cell proliferation [23]. Nevertheless, the SUCC of FOXM1 remained unexplored.

Therefore, this study aimed to investigate the effects of SH3PXD2A-AS1 on DDP resistance in NSCLC and the associated mechanism, with the goal of identifying a possible therapeutic approach for NSCLC.

## Methods and materials

### Bioinformatics analysis

The expression of SH3PXD2A-AS1 in normal and lung squamous cell carcinoma (LUSC) tissues, lung adenocarcinoma (LUAD), and normal tissues was analyzed using the Gene Expression Profiling Interactive Analysis (GEPIA) database (http://gepia.cancer-pku.cn/index.html). In addition, the effects of SH3PXD2A-AS1 and FOXM1 genes on the survival rate in NSCLC were analyzed using the GEPIA database (http://gepia.cancer-pku.cn/detail.php). The SuccinSite database (http://systbio.cau.edu.cn/SuccinSite/) was used to screen succinylation sites of FOXM1 by choosing the highest three combined random forest scores.

### Cell culture

Two NSCLC cell lines including A549 (catalog number: BNCC337696) and H1299 (catalog number: BNCC100859), and human embryonic kidney (HEK)-293T (catalog number: BNCC341976) cells were purchased from BeNa culture collection (Beijing, China). All cells were cultured with 90% Roswell Park Memorial Institute (RPMI)-1640 (Hyclone, Logan, UT, USA), 10% fetal bovine serum (FBS, Gibco, Carlsbad, CA, USA) and 1% penicillin/streptomycin mixture (Sigma, St. Louis, MO, USA). DDP resistant cell lines (A549/DDP and H1299/DDP) were purchased from Biovector National Typical Culture Center (Beijing, China) and cultured in Ham’s F-12 K medium (Pricella Life Technology Co., LTD, Wuhan, China) containing 10% FBS, 1 µmol/L DDP (Sigma) and 1% penicillin/streptomycin mixture.

### Reverse transcription-quantitative polymerase chain reaction (RT-qPCR)

TRIzol reagent (Sigma) was applied to extract total RNA from cells. Then, RNA was reverse transcribed into cDNA using the PrimeScript RT kit (Takara, Dalian, China), and the qPCR amplification experiment was performed using the SYBR Premix ExTaqII kit (Takara) with the reaction conditions: 95 °C for 10 min, 40 cycles of 95 °C for 15 s, 60 °C for 20 s, and a cycle of 72 °C for 20 s. The gene expression was calculated by the 2^−ΔΔCT^ method. Primers used in this study are listed as follows: SH3PXD2A antisense RNA 1 (SH3PXD2A-AS1), forward, 5′- CAGGAGTGTGCCACCATGCTTG-3′ and reverse, 5′-GGCAAGACTGGCTCATGAACTCTC-3′; centromere Protein F (CENPF), forward, 5′- TACAACGAGAGAGTAAGAACGC-3′ and reverse, 5′- CTACCTCCACTGACTTACTGTC-3′; forkhead box protein M1 (FOXM1), forward, 5′-GATCTGCGAGATTTTGGTACAC-3′ and reverse, 5′-CTGCAGAAGAAAGAGGAGCTAT-3′; kinesin-like family member 20 A (KIF20A), forward, 5′-GAATGTGGAGACCCTTGTTCTA-3′ and reverse, 5′-CCATCTCCTTCACAGTTAGGTT-3′; K (lysine) acetyltransferase (KAT) 2 A, forward, 5′-CAGTTTCGGCAGAGGTCTCA-3′ and reverse, 5′-ATGAGTGGTTTCGTAGCGGG-3′; KAT3B, forward, 5′-AGCCAAGCGGCCTAAACTC-3′ and reverse, 5′-TCACCACCATTGGTTAGTCCC-3′; Carnitine palmitoyltransferase 1 A (CPT1A), forward, 5′-ATCAATCGGACTCTGGAAACGG-3′ and reverse, 5′-TCAGGGAGTAGCGCATGGT-3′; sirtuin (SIRT) 5, forward, 5′-CTGGATCCTGCCATTCTGGA-3′ and reverse, 5′-CTGGGTACACCACAGAGGAA-3′; SIRT7, forward, 5′-CAGGGAGTACGTGCGGGTGT-3′ and reverse, 5′-TCGGTCGCCGCTTCCCAGTT-3′; glyceraldehyde-3-phosphate dehydrogenase (GAPDH), 5′-GACTCATGACCACAGTCCATGC-3′ and reverse, 5′-AGAGGCAGGGATGATGTTCTG-3′.

### Cell transfection

SH3PXD2A-AS1 small interfering (si) RNAs (si-SH3PXD2A-AS1#1/2), siRNA negative control (si-NC), short hairpin (sh) SH3PXD2A-AS1 (shSH3PXD2A-AS1), shNC, overexpression vectors (KAT2A, KAT3B, CPT1A, SIRT5 and SIRT7), and empty vector used in this study were purchased from GenePharma (Shanghai, China). The cells (5 × 10^5^ cells/well) were inoculated in 6-well plates (Corning, NY, USA). After the cell confluence reached 80%, transfection was performed using Lipofectamine 2000 (Invitrogen) according to the manufacturer’s instructions. The cells were transfected for 48 h, and the transfection efficiency was detected by RT-qPCR.

Flag-FOXM1-K12S, Flag-FOXM1-K259S and Flag-FOXM1-K324S were designed by Genscript Biotechnology Co., LTD (Nanjing, China). Briefly, serine mutations were introduced at K12 (K12S), K259 (K259S) and K324 (K324S) sites of FOXM1. Then, the Flag-FOXM1-WT, Flag-FOXM1-K12S, Flag-FOXM1-K259S and Flag-FOXM1-K324S plasmids were transfected into HEK-293T cells for 24 h.

### Cell counting kit-8 (CCK-8) assay

Cell viability was detected by the CCK-8 kit (Dojindo, Japan). A549, H1299, A549/DDP and H1299/DDP cell lines were seeded into 96-well plates at the density of 1 × 10^3^ cells/well. Three replicate wells were set up. The cells were treated with DDP at the dose of 1, 2, 4, 8, 16, 32, or 64 µmol/L for 48 h at 37˚C, respectively. Then, the medium with DDP were removed, and 90 µL F-12 K medium and 10 µL CCK-8 solution were added into each well of the plates to incubate for 2 h in the incubator. The 50% inhibiting concentration (IC50) value was then calculated by GraphPad Prism (v8.0.1, GraphPad Software Inc., San Diego, CA, USA). In addition, the transfected A549/DDP and H1299/DDP cell lines were maintained in the incubator for 12, 24, 48 and 72 h at 37˚C. Following incubation, 10 µL of CCK-8 solution was added to each well to incubate with cell lines for 2 h at 37˚C. The absorbance was assessed at 450 nm using a microplate reader (Synergy HT, Bio-Tek, United States).

### Flow cytometry

The apoptosis rate of A549/DDP and H1299/DDP cell lines was assessed by fluorescein isothiocyanate (FITC) and propidium iodide (PI) double staining solution using a commercial Apoptosis Detection Kit (Yeason Biotechnology Co., LTD, Shanghai, China). Transfected cells were digested with 0.25% trypsin for 1 min and collected after centrifugation. Then, the cells were washed twice with pre-cooled PBS (Thermo Fisher) and re-suspended in 100 µl of binding buffer (Thermo Fisher). Then, 5 µL of FITC and 10 µL PI were added to incubate with cells for 15 min at 37℃ in the dark. After that, 400 µL binding buffer was added to the incubate and fully mixed. Cell samples were loaded onto the FACS CantoII flow cytometer (BD Biosciences), and cell apoptosis rate was assessed by the BD FACSDiva software. Flow cytometry was conducted three times with three biological repetitions each time.

### Western blot

The cells were harvested and the lysis buffer (Thermo Fisher) was used to extract the protein. After the protein concentration was determined by the BCA method (Thermo Fisher), 50 µg of protein was separated by a 10% SDS-PAGE, and then transferred to the PVDF membrane (Thermo Fisher). The membrane was blocked using 5% skim milk for 1 h, and subsequently incubated with the primary antibodies overnight at 4 °C. The membrane was washed 3 times with Tris buffered saline Tween (TBST, Thermo Fisher), and incubated with the secondary antibody for 1 h at room temperature. Finally, an enhanced chemiluminescence solution (Pierce; Thermo Fisher) was used for protein signal detection.

### Co-immunoprecipitation (Co-IP) and IP assays

Co-IP was used to detect the interaction between FOXM1 and SIRT7 in HEK-293T cells. The cells were lysed on ice using RIPA (Beyotime Biotechnology, Shanghai, China) buffer containing protease inhibitors for 30 min. The supernatant was collected and a small amount of it was taken as the input group. FOXM1, SIRT7 or lgG antibody (Thermo Fisher, 2 µg) was added into the remaining supernatant for incubation overnight at 4 °C. Afterward, 10 µL of protein A agarose beads (Thermo Fisher) was pre-treated by washing with appropriate lysis buffer (Beyotime) for three times, and then added to the cell lysate and antibody complex, and slowly shaken at 4℃ for 2 h to make the antibody conjugated with the protein A agarose beads. After the immunoprecipitation reaction, the complex was centrifuged at 3,000 rpm for 3 min at 4 °C. Next, the supernatant was discarded and the agarose beads were washed with 1 mL of lysis buffer for three times. Finally, 15 µL of 2×SDS loading buffer (Beyotime) was added and boiled for 5 min. The precipitated protein was then analyzed using western blot assay as described above.

### RNA pull-down assay

The in vitro transcribed SH3PXD2A-AS1 was labeled using biotin (Thermo Fisher). Subsequently, streptavidin magnetic beads (Invitrogen, USA) were used to capture biotin-labeled RNA, and then the RNA-beads were incubated with the cell lysate to make the proteins bound to the target RNA attached to the magnetic beads. After washing off the unbound proteins, the RNA-protein complex was eluted from the magnetic beads by SDS-PAGE loading buffer (Thermo Fisher). RNA was extracted using the RNeasy Mini Kit (Qiagen) and SIRT7 expression was evaluated by RT-qPCR.

### RNA immunoprecipitation (RIP) assay

RIP assay was used to explore the interaction between SH3PXD2A-AS1 and SIRT7 in HEK-293T cells using the Imprint RIP Kit (Millipore, USA). In brief, The cells were washed twice with pre-cooled PBS, then re-suspended with passive lysis buffer (Promega Biotech Co., Ltd, Beijing) for 30 min at 4 °C. Then, the cell supernatant was incubated with magnetic beads bound with IgG and SIRT7 antibodies (Abcam, USA) for 4 h at 4 °C. After incubation, the beads were washed and eluted. Then, proteinase K was added to remove protein at 55 °C for 30 min. The RNAs were isolated and qPCR was performed to detect SH3PXD2A-AS1 expression.

### RNA fluorescence in situ hybridization (FISH) and immunofluorescence

Specific SH3PXD2A-AS1 probe (GenePharma Biotech, Guangzhou) was prepared and incubated at 75℃ for 5 min, and immediately placed at 0℃ for 10 min to denature the double-stranded DNA probe. HEK-293T cells were seeded into µ-Slide 8-well chamber slide (Ibidi, Martinsried, Germany). The cells were washed twice with pre-cooled PBS and fixed with 4% sterile paraformaldehyde (PFA, Thermo Fisher) at 37℃ for 15 min. Then the cells were washed with PBS twice, treated with 0.2% Triton X-100 (Thermo Fisher) at 37℃ for 15 min, and incubated with denatured probes at 37 °C overnight. Then, cells were rinsed with saline sodium citrate buffer (Thermo Fisher) in accordance with the order 4×, 2×, and 1×. After RNA FISH, cells were fixed again for 5 min in 2% formaldehyde and subjected to immunofluorescence with anti-SIRT7 primary antibody (Thermo Fisher) and fluorescent secondary antibody (Thermo Fisher) sequentially. The nuclei were counterstained with 4′,6-diamidino-2-phenylindole (DAPI, Thermo Fisher), and images were obtained on an LSM700 confocal microscope (Carl Zeiss, Oberkochen, Germany).

### Animal study

A total of 12 male BALB/c (6 weeks old) mice were purchased from Charles River (Beijing, China) and housed in cages with 24℃, a 12 h alternating light/dark cycle and free access to water and food. After one-week adaptive feeding, the mice were randomly divided into two groups (*n* = 6 per group): Lentivirus (Lv)-shNC and Lv-shSH3PXD2A-AS1 A549/DDP cells were infected with Lv-shNC and Lv-shSH3PXD2A-AS1 and adjusted cell density at 5 × 10^7^ cells/mL. The mice were subcutaneously injected with 100 µL Lv-shNC-infected or Lv-shSH3PXD2A-AS1-infected A549/DDP cells to establish a tumor-bearing mouse model. Tumor volume was measured using a vernier caliper every week and quantified using the formula: Volume (mm^3^) = (length × width^2^)/2. After the fourth measurement of tumor volume, the mice were euthanized by carbon dioxide inhalation followed by cervical dislocation. The tumors were isolated from all mice and weighed.

### Immunohistochemistry (IHC) assay

Mice tumor tissue paraffin Sect. (4 μm) were incubated with anti-Ki67 (Abcam, ab16667, 1/200), anti-SIRT7 (Abcam, ab259968, 1/100), and anti-FOXM1 (Abcam, ab207298, 1/250) at 4 °C overnight followed by incubating with the secondary antibody (Abcam, ab205719, 1/5000) at room temperature for 30 min. Then, the sections were stained with diaminobenzidine solution for 3 min at room temperature. After washing using moving water and sealing, the images were visualized under a microscope.

### Statistical analysis

The SPSS 21.0 software was used to analyze data. Data are expressed as mean ± standard deviation (SD). Student’s t-test was used for comparison between two groups. One way analysis of variance (ANOVA) was used for comparison among three groups. Statistical analyses were performed using GraphPad Prism (v8.0.1, GraphPad Software Inc., San Diego, CA, USA). *p* < 0.05 indicates that the difference is statistically significant.

## Results

### SH3PXD2A-AS1 expression was upregulated in DDP-resistant NSCLC cell lines

**Fig. 1:**
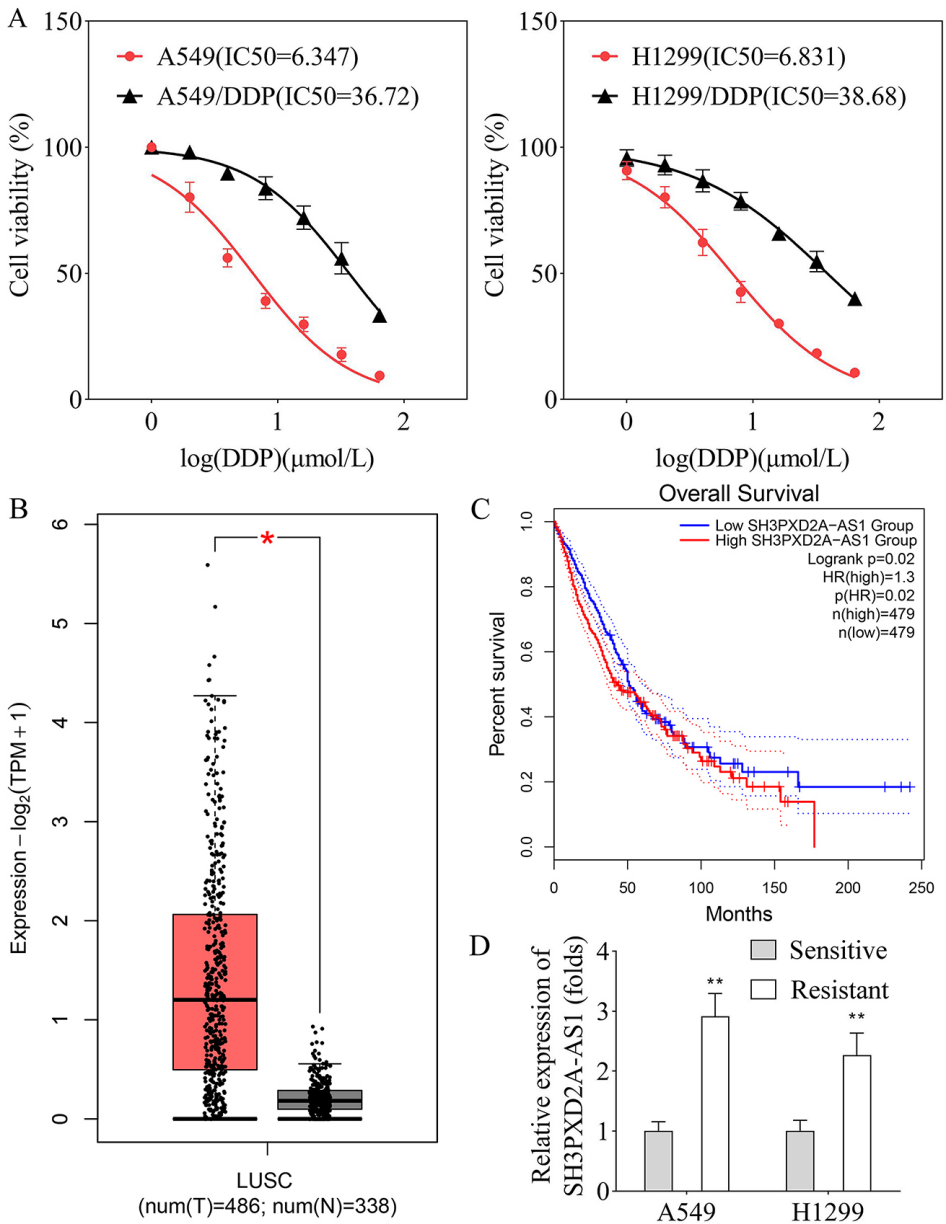
SH3PXD2A-AS1 expression was upregulated in DDP-resistant NSCLC cell lines. **A**, CCK-8 assay was performed to analyze cell viability of A549, H1299, A549/DDP, and H1299/DDP cells; **B**, SH3PXD2A-AS1 expression was analyzed in LUSC tissues (*n* = 486) and normal tissues (*n* = 338) using the GEPIA database; **C**, GEPIA database was used to analyze the survival rate of LUSC and LUAD patients with high or low SH3PXD2A-AS1 expression; **D**, The expression of SH3PXD2A-AS1 was examined in sensitive and resistant A549 and H1299 cell lines by RT-qPCR. (^*^p < 0.05; ^**^p < 0.01)

To detect the resistance of DDP in purchased A549/DDP and H1299/DDP cells, CCK-8 assay was performed to analyze the cell viability of NSCLC cells. The cell viability of A549/DDP and H1299/DDP cell lines was decreased lower than that of A549 and H1299 cells. The IC50 values for A549/DDP and H1299/DDP cell lines were higher than A549 and H1299 cell lines (Fig. 1A), suggesting the A549/DDP and H1299/DDP cell lines were resistant to DDP. Bioinformatic analysis using the GEPIA database showed the expression of SH3PXD2A-AS1 was upregulated in the LUSC tissues compared with that in normal tissues (Fig. 1B). Moreover, highly expressed SH3PXD2A-AS1 exhibited lower survival in LUAD and LUSC patients (Fig. 1C), while SH3PXD2A-AS1 expression was upregulated in A549 and H1299 resistant cell lines compared with A549 and H1299 sensitive cell lines (Fig. 1D), implying the important role of SH3PXD2A-AS1 in DDP-resistance in NSCLC.

### Silencing of SH3PXD2A-AS1 decreased cell viability and increased apoptosis of A549/DDP and H1299/DDP cell lines

**Fig. 2:**
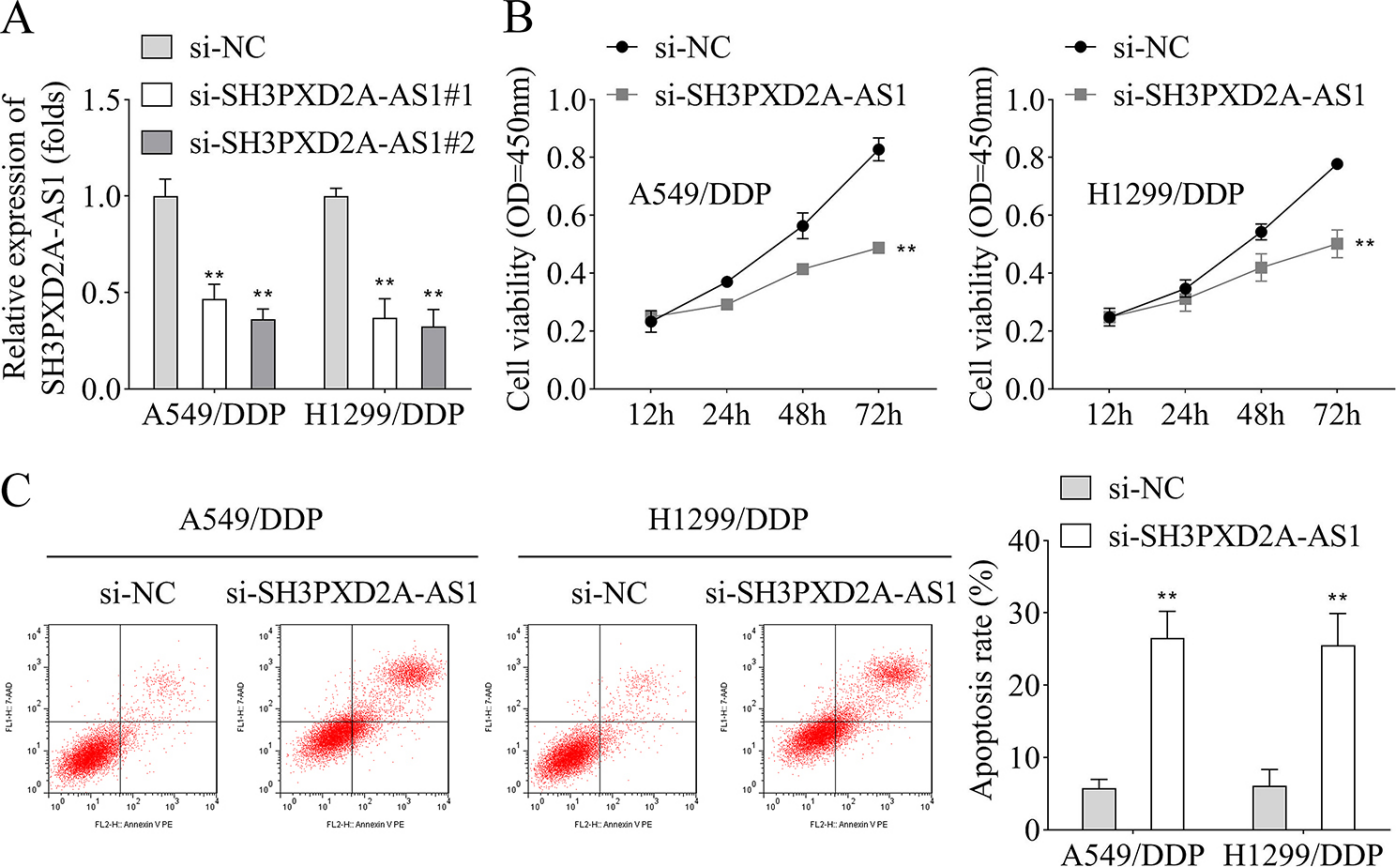
Silencing of SH3PXD2A-AS1 decreased cell viability and promoted apoptosis of A549/DDP and H1299/DDP cell lines. **A**, The transfection efficiency of si-SH3PXD2A-AS1#1/2 in A549/DDP and H1299/DDP cell lines was detected by RT-qPCR; **B**, CCK-8 assay was performed to assess the cell viability of A549/DDP and H1299/DDP cell lines when SH3PXD2A-AS1 was silenced; **C**, Flow cytometry was performed to detect the apoptosis rate of A549/DDP and H1299/DDP cell lines after SH3PXD2A-AS1 silencing. (^**^p < 0.01)

Si-SH3PXD2A-AS1#1/2 was transfected into A549/DDP and H1299/DDP cell lines to further investigate the effects of SH3PXD2A-AS1 in DDP resistance development, and results showed downregulated SH3PXD2A-AS1 expression (Fig. 2A). Moreover, CCK-8 assay and flow cytometry results indicated that SH3PXD2A-AS1 knockdown suppressed the cell viability (Fig. 2B), and induced apoptosis in A549/DDP and H1299/DDP cell lines (Fig. 2C).

### Silencing of SH3PXD2A-AS1 increased the SUCC level of FOXM1 in A549/DDP and H1299/DDP cell lines

**Fig. 3:**
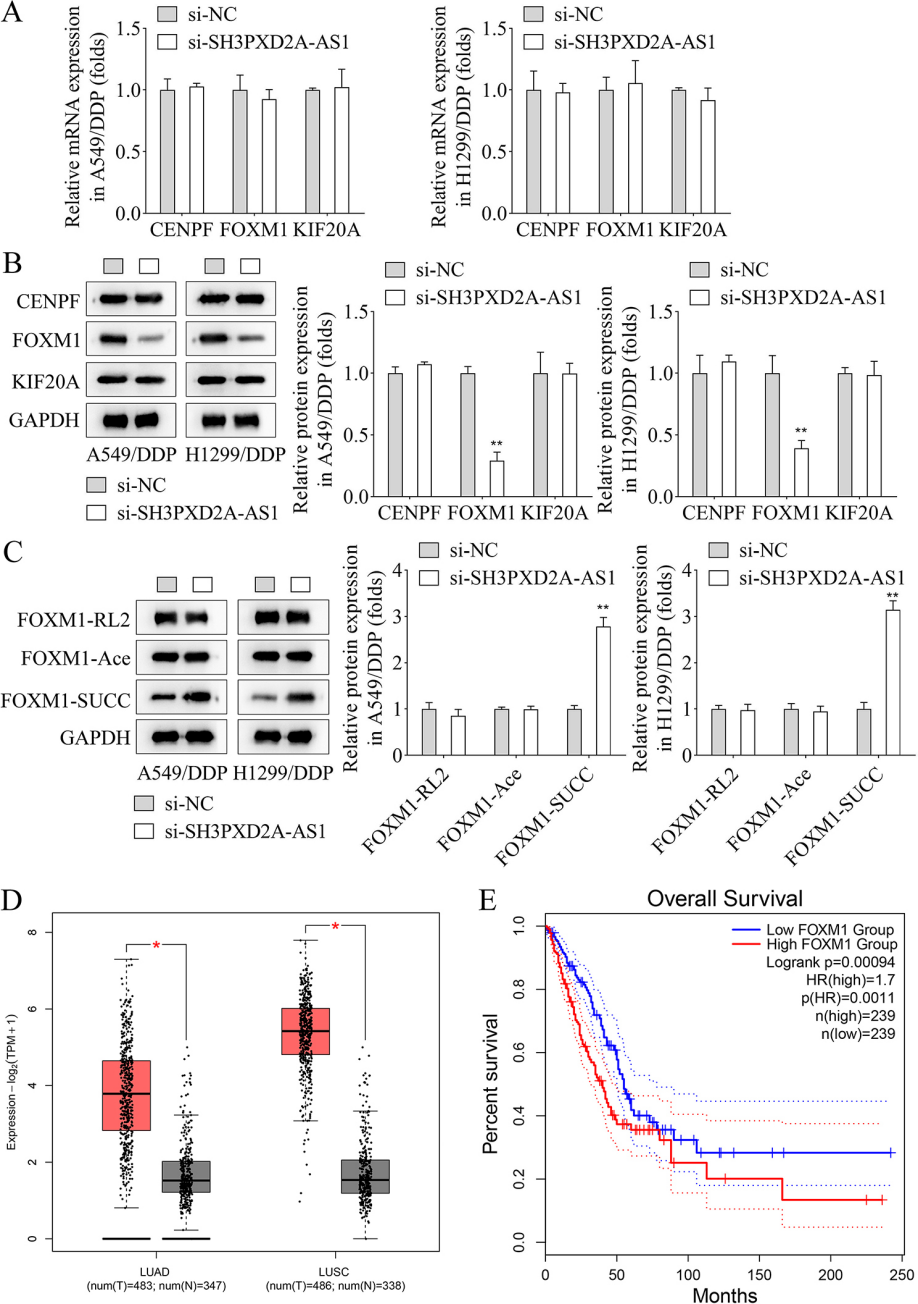
Silencing of SH3PXD2A-AS1 promoted the succinylation level of FOXM1 in A549/DDP and H1299/DDP cell lines. **A**, RT-qPCR was used to detect the expression of CENPF, FOXM1 and KIF20A in A549/DDP and H1299/DDP cell lines after SH3PXD2A-AS1 silencing; **B**, The protein levels of CENPF, FOXM1 and KIF20A in A549/DDP and H1299/DDP cell lines were detected by western blot when the SH3PXD2A-AS1 was silenced; **C**, Western blot was performed to assess the glycosylation (RL2), acetylation (Ace) and succinylation (SUCC) modification of FOXM1 after SH3PXD2A-AS1 silencing in A549/DDP and H1299/DDP cell lines; **D**, The FOXM1 expression was analyzed in LUAD or LUSC tissues and normal tissues in the GEPIA database; **E**, GEPIA database was used to analyze the effects of the FOXM1 on the survival rate of NSCLC patients. (^*^p < 0.05; ^**^p < 0.01)

Expression of genes associated with SH3PXD2A-AS1 was evaluated, and results showed that CENPF, FOXM1, and KIF20A mRNA expression remained unaffected by SH3PXD2A-AS1 knockdown in A549/DDP and H1299/DDP cell lines (Fig. 3A). Silencing of SH3PXD2A-AS1 decreased the protein level of FOXM1, while that of CENPF and KIF20A were not altered in A549/DDP and H1299/DDP cell lines (Fig. 3B). Moreover, knockdown of SH3PXD2A-AS1 significantly enhanced the level of FOXM1-SUCC in A549/DDP and H1299/DDP cell lines, while the FOXM1 acetylation (Ace) and glycosylation (RL2) levels remained unaffected (Fig. 3C). FOXM1 expression in LUAD or LUSC and normal tissues was identified using the GEPIA database, and results showed that the expression of FOXM1 was elevated in LUAD or LUSC tissues in comparison to normal tissues (Fig. 3D), in addition to highly expressed FOXM1 showing reduced survival of NSCLC (Fig. 3E).

### FOXM1 was succinylated at K259 site mediated by SIRT7

**Fig. 4:**
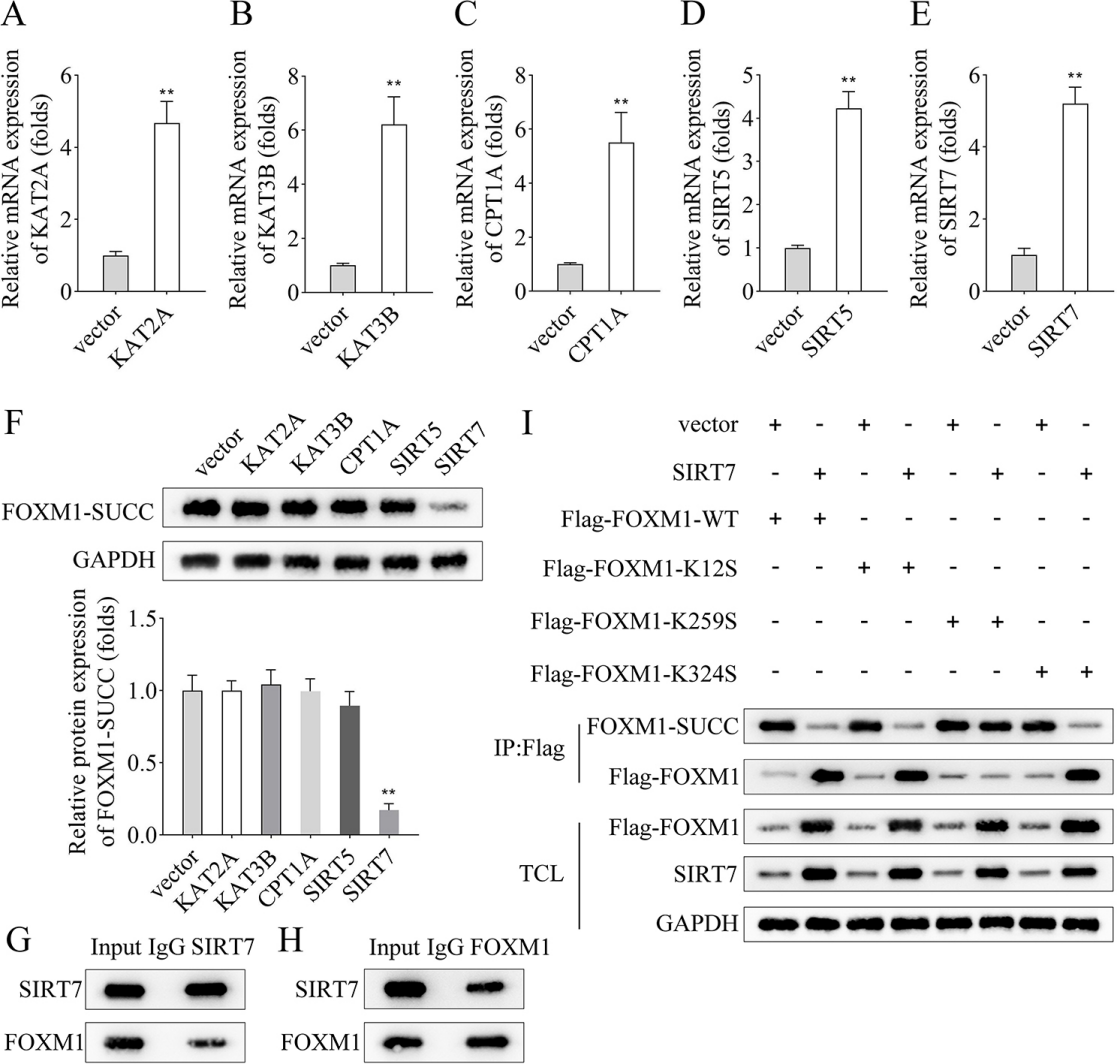
FOXM1 was succinylated at K259 site mediated by SIRT7. The transfection efficiency of **A**, KAT2A, **B**, KAT3B, **C**, CPT1A, **D**, SIRT5 and **E**, SIRT7 overexpression plasmids in HEK-293T cells was detected by RT-qPCR; **F**, The HEK-293T cells transfected with KAT2A, KAT3B, CPT1A, SIRT5 and SIRT7 overexpression vectors and empty vector, and the level of FOXM1-SUCC was assayed by western blot; **G-H**, Co-IP assay was used to analyze the interaction between FOXM1 and SIRT7 in HEK-293T cells; **I**, IP followed by western blot assay was used to analyze the succinylation site of FOXM1. (^**^p < 0.01)

Overexpressed plasmids of KAT2A, KAT3B, CPT1A, SIRT5, and SIRT7 were transfected into HEK-293T cells to investigate which enzyme regulated FOXM1 SUCC, and the results showed that their expression was significantly upregulated (Fig. 4A-E). Similarly, western blot results indicated that overexpressing of SIRT7 decreased FOXM1-SUCC level, which remained unaltered post KAT2A, KAT3B, CPT1A, and SIRT5 overexpression (Fig. 4F). Co-IP assay results revealed interaction of FOXM1 with SIRT7 in HEK-293T cells (Fig. 4G and H). We searched the SuccinSite website and screened out three possible FOXM1 SUCC sites, K12, K259, and K324, followed by introducing serine mutations were introduced at these three sites. The IP results showed that overexpressing of SIRT7 reduced the FOXM1-SUCC level and increased the FOXM1 protein level. Additionally, SIRT7 inhibited SUCC and increased the protein level of FOXM1 when K12 and K324 sites were mutated, whereas FOXM1-SUCC and FOXM1 protein levels remained unaffected post-K259 site mutation (Fig. 4I).

### Overexpressing of SIRT7 reversed the increased FOXM1-SUCC level induced by silencing of SH3PXD2A-AS1 in HEK-293T cells

**Fig. 5:**
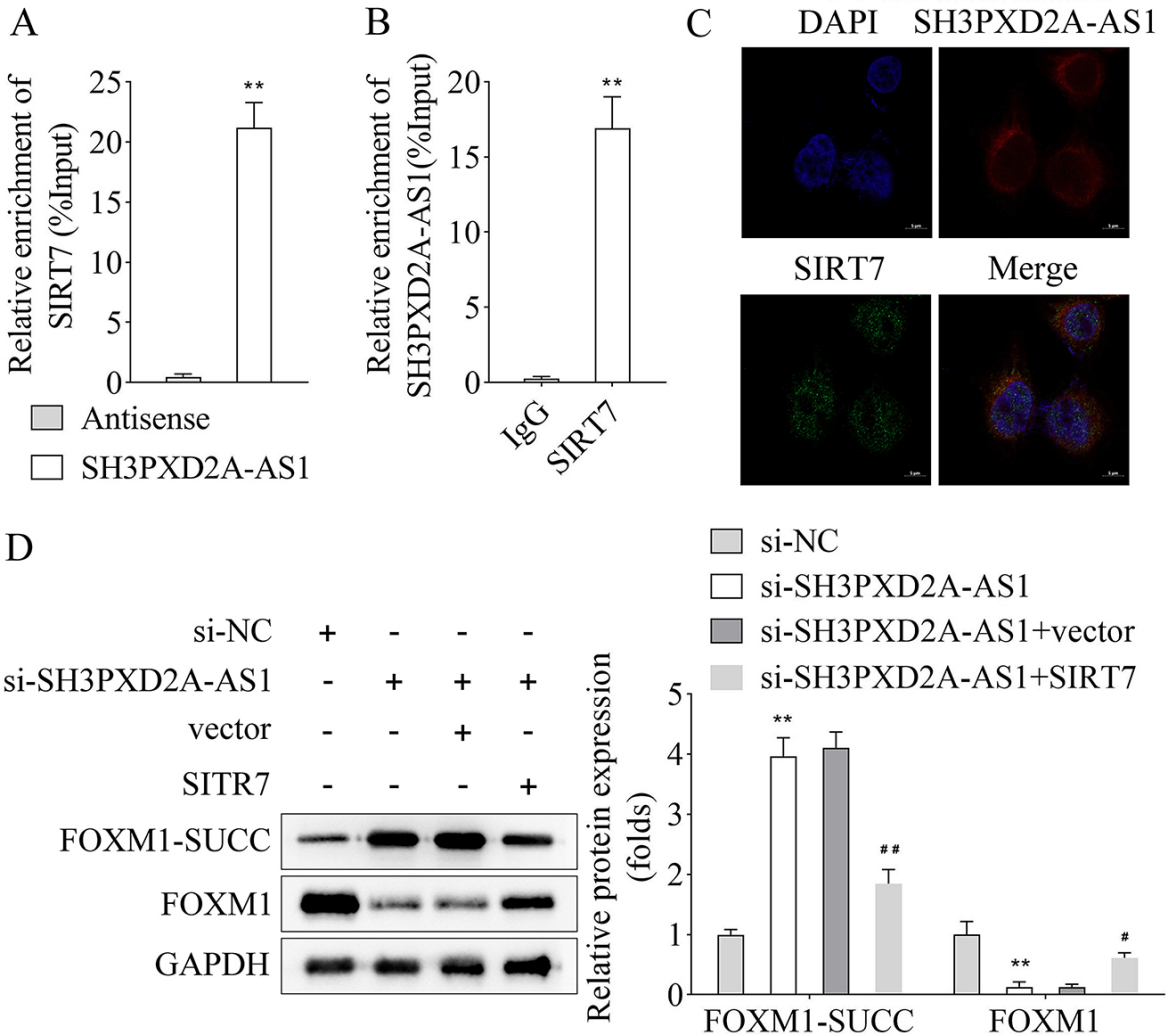
Overexpressing of SIRT7 reversed the increased FOXM1-SUCC level in HEK-293T cells induced by silencing SH3PXD2A-AS1. **A**, RNA pull-down assay was performed to test the interaction of SH3PXD2A-AS1 and SIRT7; **B**, RIP assay was used to detect the interaction of SH3PXD2A-AS1 and SIRT7; **C**, RNA FISH and immunofluorescence assays showed the subcellular location of SH3PXD2A-AS1 and SIRT7. Nuclei were stained with DAPI. Scale bar = 5 μm; **D**, The protein level of FOXM1 and FOXM1-SUCC in each group in HEK-293T cells were detected by western blot. (^**^p < 0.01 vs. antisense or si-NC group; ^#^*p* < 0.05, ^##^*p* < 0.01 vs. si-SH3PXD2A-AS1 + vector group)

RNA pull-down and RIP assays cemented the interaction between SH3PXD2A-AS1 and SIRT7 in HEK-293T cells (Fig. 5A and B). Moreover, RNA FISH and immunofluorescence assay showed that SH3PXD2A-AS1 colocalized with SIRT7 in the cytoplasm, indicating the direct binding of SH3PXD2A-AS1 with SIRT7 (Fig. 5C). Similarly, SH3PXD2A-AS1 silencing enhanced the FOXM1-SUCC level and decreased FOXM1 protein levels, which were reversed by SIRT7 overexpression (Fig. 5D).

### Overexpression of SIRT7 reversed cell viability and apoptosis induced by silencing of SH3PXD2A-AS1 in A549/DDP and H1299/DDP cell lines

**Fig. 6:**
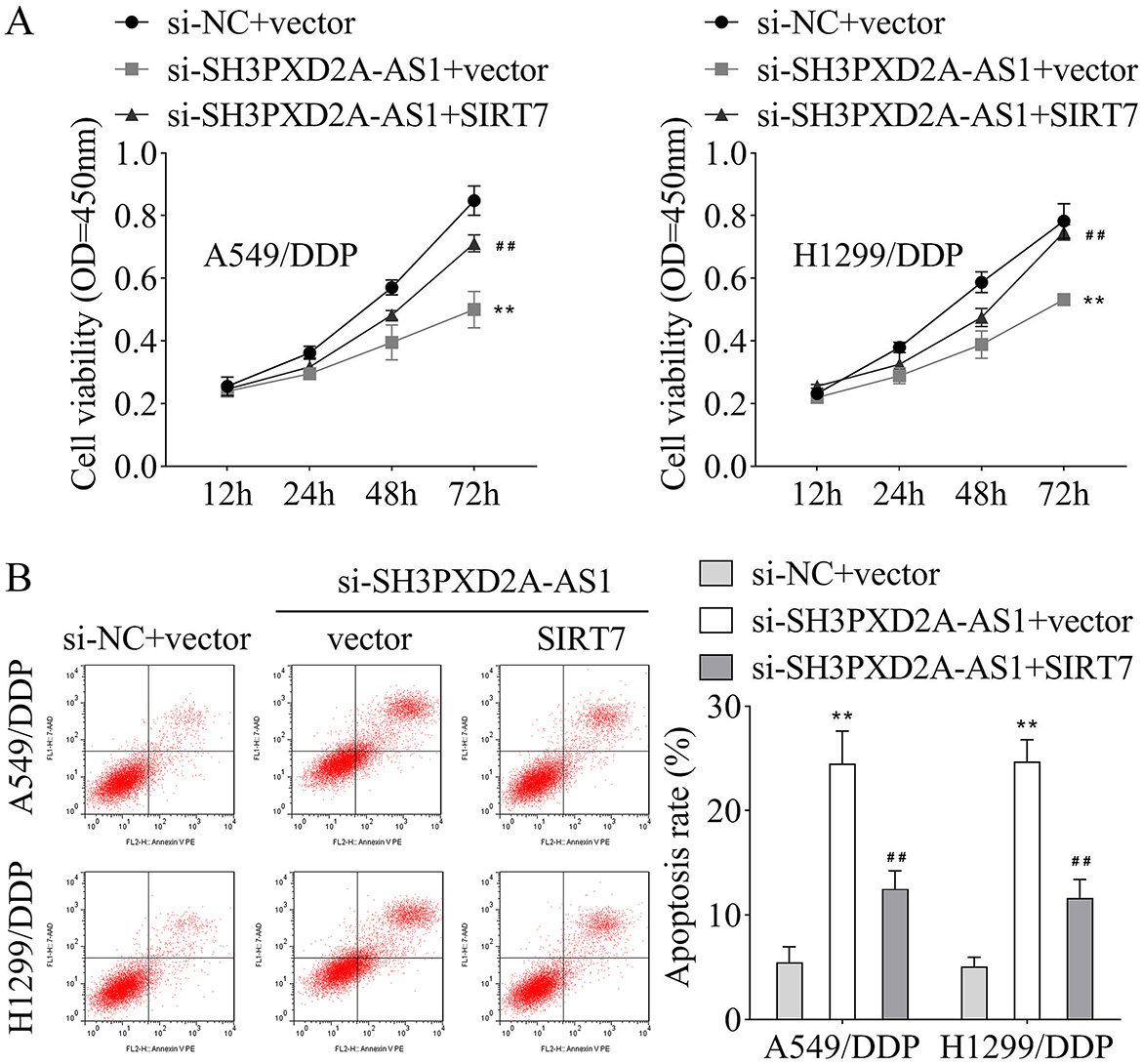
Overexpression of SIRT7 reversed cell viability and apoptosis induced by silencing SH3PXD2A-AS1 in A549/DDP and H1299/DDP cell lines. **A**, CCK-8 assay was performed to assess the cell viability of A549/DDP and H1299/DDP cell lines after SH3PXD2A-AS1 silencing and SIRT7 overexpressing; **B**, Flow cytometry was performed to detect the apoptosis rate of A549/DDP and H1299/DDP cell lines after SH3PXD2A-AS1 silencing and SIRT7 overexpressing. (^**^p < 0.01 vs. si-NC + vector group; ^##^*p* < 0.01 vs. si-SH3PXD2A-AS1 + vector group)

Si-SH3PXD2A-AS1 and SIRT7 overexpression plasmids were transfected into A549/DDP and H1299/DDP cell lines to investigate further the effects of SH3PXD2A-AS1 and SIRT7 on cell viability and apoptosis. Results indicated that silencing of si-SH3PXD2A-AS1 suppressed cell viability, while overexpressing of SIRT7 restored the inhibited cell viability in A549/DDP and H1299/DDP cell lines (Fig. 6A). Moreover, the apoptosis rate was increased when SH3PXD2A-AS1 was silenced in A549/DDP and H1299/DDP cell lines, which were partly blocked by SIRT7 overexpression (Fig. 6B).

### Silencing of SH3PXD2A-AS1 suppressed tumor growth

**Fig. 7:**
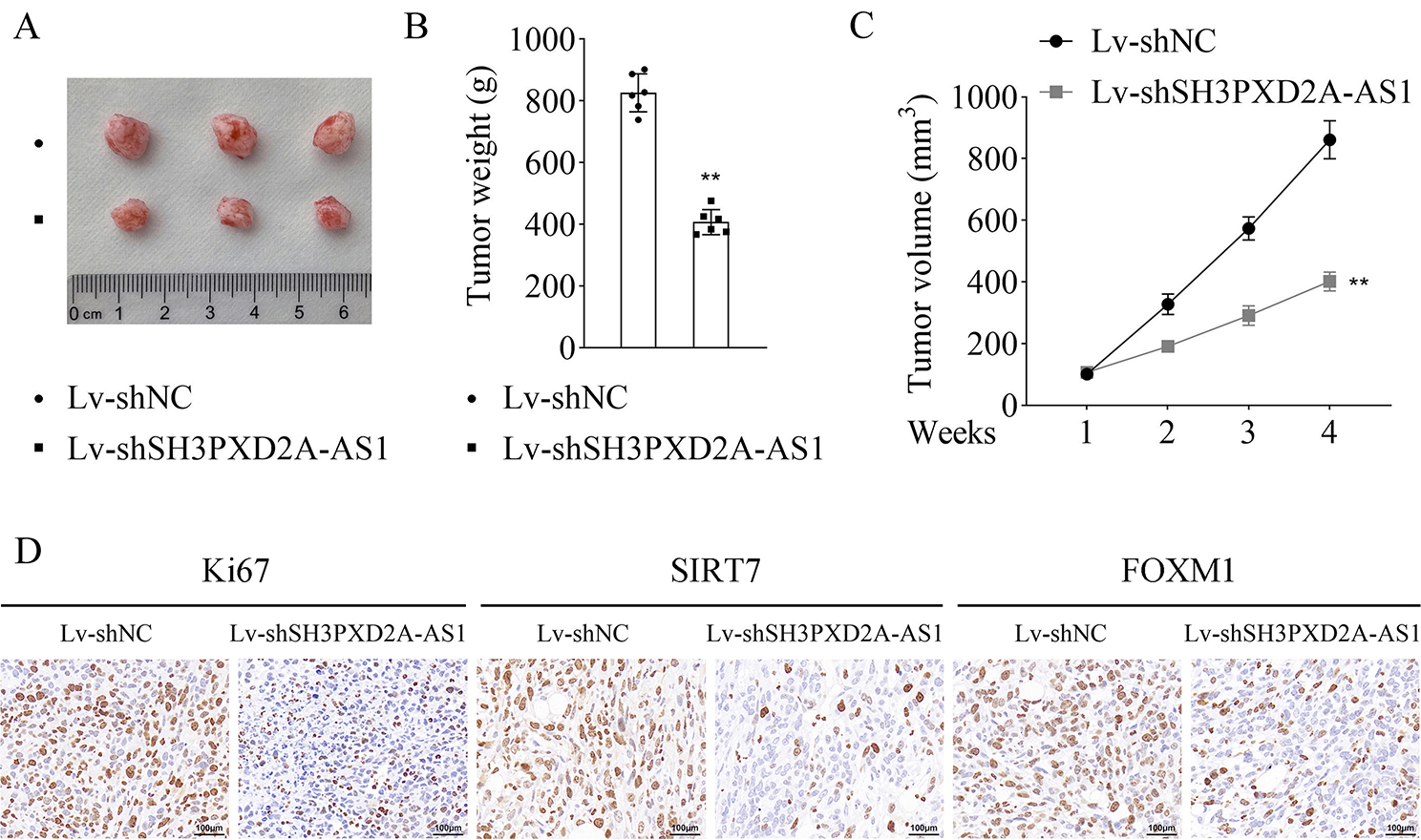
Silencing of SH3PXD2A-AS1 suppressed tumor growth. **A**, The represent images of tumors from mice of each group; **B**, Tumor weight of each group; **C**, Tumor volume was measured weekly; **D**, IHC was used to assess Ki67, SIRT7, and FOXM1 protein levels in tumors. Scale bar = 100 μm. (^**^p < 0.01)

Finally, we established the tumor-bearing mouse model to explore the role of SH3PXD2A-AS1 in vivo. The results showed that SH3PXD2A-AS1 inhibition reduced tumor size, weight, and volume compared with the Lv-shNC group (Fig. 7A-C). Moreover, IHC results indicated that the protein levels of Ki67, SIRT7, and FOXM1 were downregulated after SH3PXD2A-AS1 inhibition (Fig. 7D).

## Discussion

This study results revealed that expression of SH3PXD2A-AS1 was upregulated in DDP-resistant NSCLC cell line, whereas recent studies have also shown its upregulation in NSCLC and colorectal cancer [2, 10]. The occurrence and progression of NSCLC are often accompanied by infinite cell proliferation [24]. To further explore the biological effects of SH3PXD2A-AS1 on DDP resistance, results of knockdown experiments showed that silencing SH3PXD2A-AS1 in A549/DDP and H1299/DDP cell lines suppressed the cell viability and elevated apoptosis, implying that lack of SH3PXD2A-AS1 made DDP-resistant NSCLC cells more sensitive to DDP. Similarly, SH3PXD2A-AS1 has been found to promote cell proliferation and accelerate cell cycle in NSCLC in vitro [2]. Although SH3PXD2A-AS1’s impact on DDP-resistant NSCLC has not been identified before; however, research indicates that resistance to DDP commonly develops in cancer patients undergoing DDP-based chemotherapy, but the sensitivity to DDP can be regained by regulating specific genes or protein modifications [25–27]. Hence, we then investigated the SH3PXD2A-AS1 regulated potential mechanism of DDP resistance in NSCLC.

According to a previous study, the mRNA expression of CENPF, KIF20A, and FOXM1 is associated with the silencing of SH3PXD2A-AS1 [2]. Our results showed insignificant differences in mRNA expressions of CENPF, KIF20A, and FOXM1, while the FOXM1 protein level was decreased after SH3PXD2A-AS1 inhibition in A549/DDP and H1299/DDP cell lines. Similarly, FOXM1 is mainly expressed in highly proliferative cells, including tumor cells [28]. In addition, FOXM1 knockdown has been reported to inhibit cell proliferation, migration, and metastasis of tumor cells [29, 30]. Similar to our findings, the expression of FOXM1 is strongly linked to resistance to DDP-based chemotherapy in patients with advanced NSCLC [31]. In addition, FOXM1 enhances the DDP resistance sensitivity in gastric and endometrial cancers [32, 33]. Results of SUCC, Ace, and glycosylation levels (RL2) of FOXM1 showed that SH3PXD2A-AS1 silencing only inhibited the FOXM1-SUCC level. The FOXM1 SUCC has not been researched, while a previous study indicates that FOXM1 protein can be modified by other post-translational modifications, including phosphorylation, ubiquitination, Ace, and methylation, which may have activator or inhibitory effects on the tumor progression [21]. Additionally, overexpressing SIRT7 was found to reduce FOXM1-SUCC level, which was consistent with a previous study indicating that SIRT7 exerts suppressive effects on SUCC [34]. SIRT7 was found to interact with FOXM1 in HEK-293T cells while overexpressing SIRT7 reversed the cell viability and apoptosis induced by post-SH3PXD2A-AS1 knockdown in our study. Similarly, SIRT7 has always been shown to have inhibitory effects on proliferation and migration, and promotive effects on apoptosis in tumor cells [35–37]. The above results implied that SH3PXD2A-AS1 interacted with SIRT7 to suppress the SUCC of FOXM1, thereby accelerating the DDP resistance of NSCLC cells. In further rescue experiments, we found that overexpression of SIRT7 reversed cell viability and apoptosis induced by silencing of SH3PXD2A-AS1 in A549/DDP and H1299/DDP cell lines. The relationship between SIRT7 and DDP resistance in NSCLC has not been found. Similarly, the sensitivity of endometrial cancer cells to DDP treatment is enhanced after SIRT7 inhibition [38]. A previous study indicates that SIRT7 deficiency ameliorates DDP-induced acute kidney injury by modulating inflammatory response [39]. Moreover, the results of our study showed that K259 was the SUCC site of FOXM1, which has not been reported before. In tumor-bearing mice, silencing of SH3PXD2A-AS1 suppressed tumor growth and the protein levels of Ki67, SIRT7, and FOXM1. These findings were in line with the results obtained from in vitro experiments. The findings of this study could provide a reference for further investigating the specific mechanism of FOXM1 in NSCLC or other cancers. The potential limitation of this study was that the underlying mechanism of DDP resistance in NSCLC may be complex, including but not limited to FOXM1 succinylation. We will further study this in our future work.

In summary, SH3PXD2A-AS1 promoted resistance to DDP in NSCLC by upregulating SIRT7 through the inhibition of FOXM1 SUCC. These findings could offer a novel perspective for the clinical treatment of DDP-resistant NSCLC.

### Electronic supplementary material

Below is the link to the electronic supplementary material.

Supplementary Material 1

## Data Availability

The datasets used and/or analysed during the current study are available from the corresponding author on reasonable request.

## Abbreviations

CCK-8: Cell counting kit-8
CENPF: Centromere protein F
CPT1A: Carnitine palmitoyl transferase 1 A
Co-IP: Co-immunoprecipitation
DAPI: 4′,6-diamidino-2-phenylindole
DDP: Cisplatin
FISH: Fluorescence in situ hybridization
FOXM1: Forkhead box M1
GEPIA: Gene Expression Profiling Interactive Analysis
HEK: Human embryonic kidney
IC50: 50% inhibiting concentration
IHC: Immunohistochemical
KIF: Kinesin-like family member
LUAD: Lung adenocarcinoma
LUSC: Lung squamous cell carcinoma
NSCLC: Non-small cell lung cancer
RIP: RNA immunoprecipitation
RT-qPCR: Reverse transcription-quantitative polymerase chain reaction
SH3PXD2A-AS1: SH3PXD2A antisense RNA 1
shRNA: Short hairpin RNA
siRNA: Small interfering RNA
SIRT: Sirtuin
SUCC: Succinylation

## References

[1] Chen P, Liu Y, Wen Y, Zhou C (2022). Non-small cell lung cancer in China. Cancer Commun (Lond).

[2] Zhou Y, Yong H, Cui W, Chu S, Li M, Li Z (2022). Long noncoding RNA SH3PXD2A-AS1 promotes NSCLC proliferation and accelerates cell cycle progression by interacting with DHX9. Cell Death Discov.

[3] Molina JR, Yang P, Cassivi SD, Schild SE, Adjei AA (2008). Non-small cell lung cancer: epidemiology, risk factors, treatment, and survivorship. Mayo Clin Proc.

[4] Cruz-Bermudez A, Laza-Briviesca R, Vicente-Blanco RJ, Garcia-Grande A, Coronado MJ, Laine-Menendez S (2019). Cisplatin resistance involves a metabolic reprogramming through ROS and PGC-1alpha in NSCLC which can be overcome by OXPHOS inhibition. Free Radic Biol Med.

[5] Xing C, Sun SG, Yue ZQ, Bai F (2021). Role of lncRNA LUCAT1 in cancer. Biomed Pharmacother.

[6] Da RS, Boeva V, Escamilla-Del-Arenal M, Ancelin K, Granier C, Matias NR (2014). Jarid2 is implicated in the initial Xist-Induced Targeting of PRC2 to the inactive X chromosome. Mol Cell.

[7] Chen D, Zhang Z, Mao C, Zhou Y, Yu L, Yin Y (2014). ANRIL inhibits p15(INK4b) through the TGFbeta1 signaling pathway in human esophageal squamous cell carcinoma. Cell Immunol.

[8] Cai R, Sun Y, Qimuge N, Wang G, Wang Y, Chu G (2018). Adiponectin AS lncRNA inhibits adipogenesis by transferring from nucleus to cytoplasm and attenuating adiponectin mRNA translation. Biochim Biophys Acta Mol Cell Biol Lipids.

[9] Hu G, Lou Z, Gupta M. The long non-coding RNA GAS5 cooperates with the eukaryotic translation initiation factor 4E to regulate c-Myc translation. PLoS ONE 2014;9: e107016. 10.1371/journal.pone.0107016 PMC4157848 25197831

[10] Hou P, Lin T, Meng S, Shi M, Chen F, Jiang T (2021). Long noncoding RNA SH3PXD2A-AS1 promotes colorectal cancer progression by regulating p53-mediated gene transcription. Int J Biol Sci.

[11] Li N, Zhan X (2019). Identification of clinical trait-related lncRNA and mRNA biomarkers with weighted gene co-expression network analysis as useful tool for personalized medicine in ovarian cancer. EPMA J.

[12] Ma Z, Peng P, Zhou J, Hui B, Ji H, Wang J (2018). Long non-coding RNA SH3PXD2A-AS1 promotes cell progression partly through epigenetic silencing P57 and KLF2 in Colorectal Cancer. Cell Physiol Biochem.

[13] Lu K, Han D (2022). A review of the mechanism of succinylation in cancer. Med (Baltim).

[14] Chen XF, Tian MX, Sun RQ, Zhang ML, Zhou LS, Jin L et al. SIRT5 inhibits peroxisomal ACOX1 to prevent oxidative damage and is downregulated in liver cancer. EMBO Rep 2018;19. 10.15252/embr.201745124 PMC5934778 29491006

[15] Wang C, Zhang C, Li X, Shen J, Xu Y, Shi H (2019). CPT1A-mediated succinylation of S100A10 increases human gastric cancer invasion. J Cell Mol Med.

[16] Chang HC, Guarente L (2014). SIRT1 and other sirtuins in metabolism. Trends Endocrinol Metab.

[17] Serrano L, Martinez-Redondo P, Marazuela-Duque A, Vazquez BN, Dooley SJ, Voigt P (2013). The tumor suppressor SirT2 regulates cell cycle progression and genome stability by modulating the mitotic deposition of H4K20 methylation. Genes Dev.

[18] Sun C, Zeng X, Guo H, Wang T, Wei L, Zhang Y (2020). MicroRNA-125a-5p modulates radioresistance in LTEP-a2 non-small cell lung cancer cells by targeting SIRT7. Cancer Biomark.

[19] Ashraf N, Zino S, Macintyre A, Kingsmore D, Payne AP, George WD (2006). Altered sirtuin expression is associated with node-positive breast cancer. Br J Cancer.

[20] Smestad J, Erber L, Chen Y, Maher LR (2018). Chromatin succinylation correlates with active gene expression and is perturbed by defective TCA cycle metabolism. iScience.

[21] Liao GB, Li XZ, Zeng S, Liu C, Yang SM, Yang L (2018). Regulation of the master regulator FOXM1 in cancer. Cell Commun Signal.

[22] Sun W, Li Y, Ma D, Liu Y, Xu Q, Cheng D (2022). ALKBH5 promotes lung fibroblast activation and silica-induced pulmonary fibrosis through miR-320a-3p and FOXM1. Cell Mol Biol Lett.

[23] Madhi H, Lee JS, Choi YE, Li Y, Kim MH, Choi Y (2022). FOXM1 inhibition enhances the therapeutic outcome of Lung Cancer Immunotherapy by modulating PD-L1 expression and cell proliferation. Adv Sci (Weinh).

[24] Yin D, Lu X, Su J, He X, De W, Yang J (2018). Long noncoding RNA AFAP1-AS1 predicts a poor prognosis and regulates non-small cell lung cancer cell proliferation by epigenetically repressing p21 expression. Mol Cancer.

[25] Xie H, Yao J, Wang Y, Ni B (2022). Exosome-transmitted circVMP1 facilitates the progression and cisplatin resistance of non-small cell lung cancer by targeting miR-524-5p-METTL3/SOX2 axis. Drug Deliv.

[26] Fu D, Wang C, Yu L, Yu R (2021). Induction of ferroptosis by ATF3 elevation alleviates cisplatin resistance in gastric cancer by restraining Nrf2/Keap1/xCT signaling. Cell Mol Biol Lett.

[27] Tang Z, He J, Zou J, Yu S, Sun X, Qin L (2021). Cisplatin-resistant HepG2 cell-derived exosomes transfer cisplatin resistance to cisplatin-sensitive cells in HCC. PeerJ.

[28] Nandi D, Cheema PS, Jaiswal N, Nag A (2018). FoxM1: repurposing an oncogene as a biomarker. Semin Cancer Biol.

[29] Zhong S, Zhou A, Qi F, Li Z, Yu Z, Lu Y (2017). Downregulating forkhead box M1 inhibits proliferation by inhibiting autophagy in the sw480 cell line. Biomed Rep.

[30] Yu C, Chen L, Yie L, Wei L, Wen T, Liu Y (2015). Targeting FoxM1 inhibits proliferation, invasion and migration of nasopharyngeal carcinoma through the epithelial–to-mesenchymal transition pathway. Oncol Rep.

[31] Wang Y, Wen L, Zhao SH, Ai ZH, Guo JZ, Liu WC (2013). FoxM1 expression is significantly associated with cisplatin-based chemotherapy resistance and poor prognosis in advanced non-small cell lung cancer patients. Lung Cancer.

[32] Li X, Liang J, Liu YX, Wang Y, Yang XH, Luan BH (2016). miR-149 reverses cisplatin resistance of gastric cancer SGC7901/DDP cells by targeting FoxM1. Pharmazie.

[33] Jiang J, Zhu J, Qiu P, Ni J, Zhu W, Wang X (2023). HNRNPA2B1-mediated m6A modification of FOXM1 promotes drug resistance and inhibits ferroptosis in endometrial cancer via regulation of LCN2. Funct Integr Genomics.

[34] Li L, Shi L, Yang S, Yan R, Zhang D, Yang J (2016). SIRT7 is a histone desuccinylase that functionally links to chromatin compaction and genome stability. Nat Commun.

[35] Tang X, Li G, Su F, Cai Y, Shi L, Meng Y (2020). HDAC8 cooperates with SMAD3/4 complex to suppress SIRT7 and promote cell survival and migration. Nucleic Acids Res.

[36] Mu P, Liu K, Lin Q, Yang W, Liu D, Lin Z (2019). Sirtuin 7 promotes glioma proliferation and invasion through activation of the ERK/STAT3 signaling pathway. Oncol Lett.

[37] Matsushima S, Sadoshima J (2015). The role of sirtuins in cardiac disease. Am J Physiol Heart Circ Physiol.

[38] Mao S, Ma J, Yu H (2019). Sirtuin-7 knockdown inhibits the growth of endometrial cancer cells by inducing apoptosis via the NF-kappaB signaling pathway. Oncol Lett.

[39] Miyasato Y, Yoshizawa T, Sato Y, Nakagawa T, Miyasato Y, Kakizoe Y (2018). Sirtuin 7 Deficiency ameliorates cisplatin-induced acute kidney Injury through Regulation of the inflammatory response. Sci Rep.

