# Supplementary material for: LncRNA SH3PXD2A-AS1 facilitates cisplatin resistance in non-small cell lung cancer by regulating FOXM1 succinylation
Source: BMC Cancer. 2024 Jul 17;24:848. doi: 10.1186/s12885-024-12624-9 (PMC11256434; doi:10.1186/s12885-024-12624-9)
Supplement: Supplementary file 1 — Supplementary Material 1 [file 12885_2024_12624_MOESM1_ESM.pdf]

marker
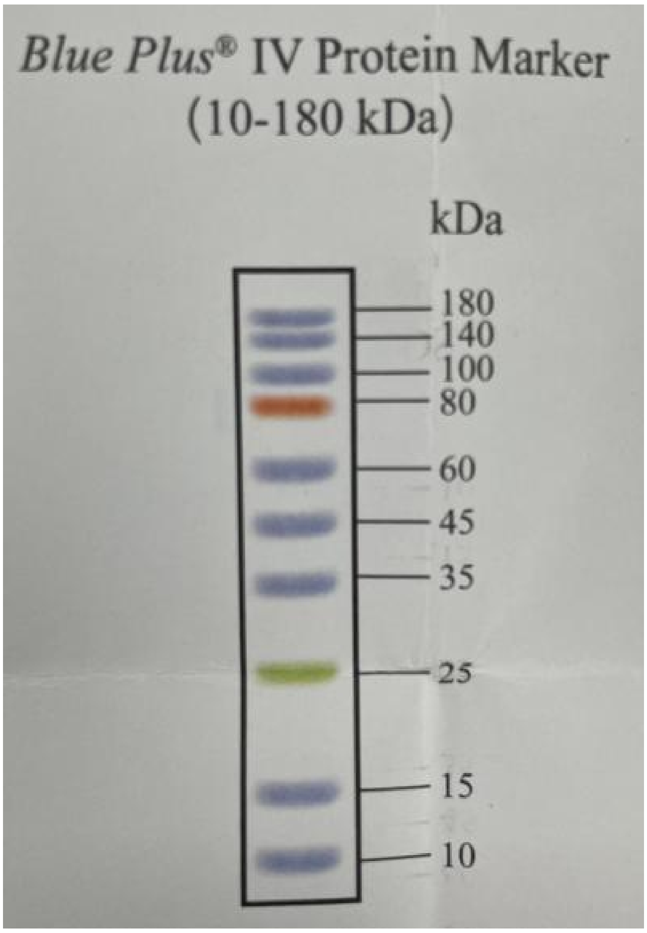 *Blue Plus*® IV Protein Marker<br>(10-180 kDa)<br>kDa<br>180<br>140<br>100<br>80<br>60<br>45<br>35<br>25<br>15<br>10

Figure3B	A549/DDP		H1299/DDP	
	si-NC	si-SH3PXD2A-AS1	si-NC	si-SH3PXD2A-AS1
CENPF	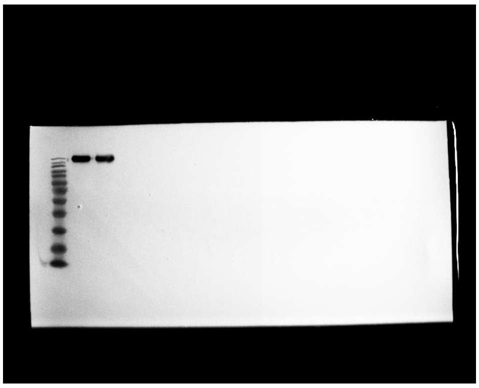		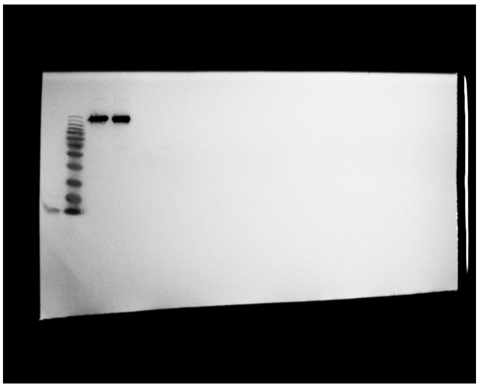	
FOXM1	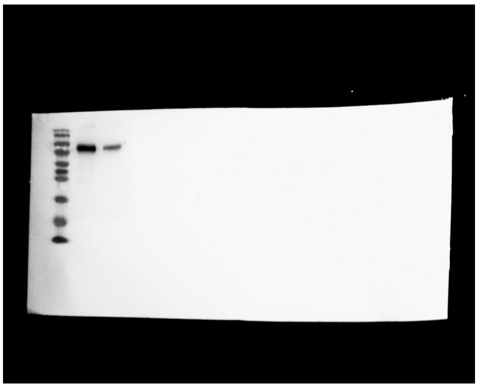		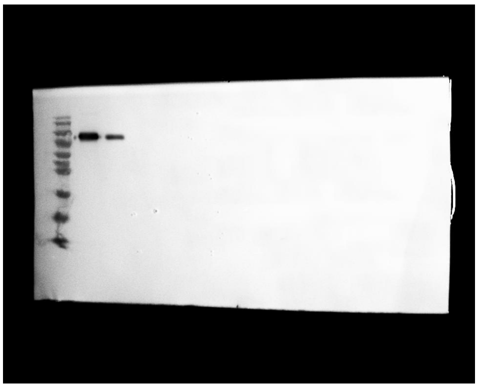	
KIF20A	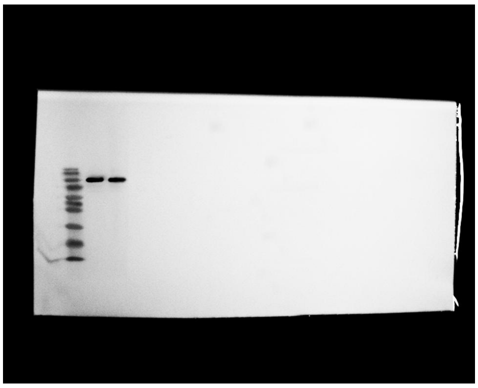		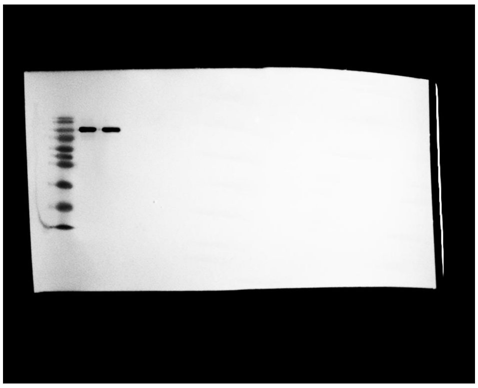	
GAPDH	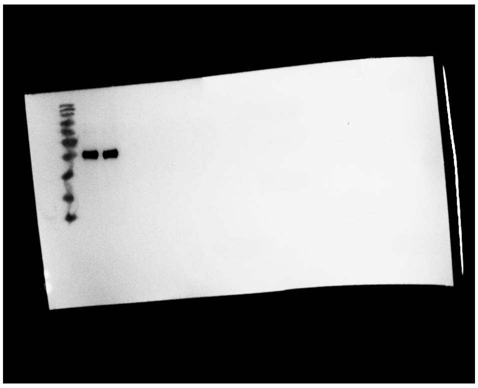		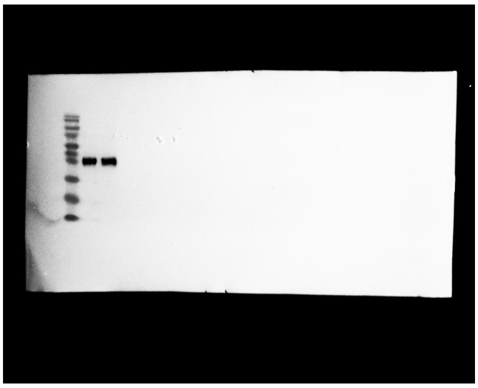	

Figure3C	A549/DDP		H1299/DDP	
	si-NC	si-SH3PXD2A-AS1	si-NC	si-SH3PXD2A-AS1
FOXM1-RL2	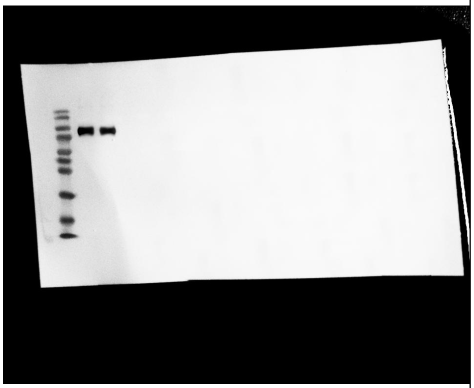		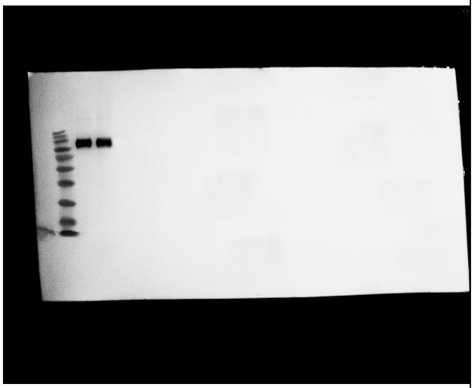	
FOXM1-Ace	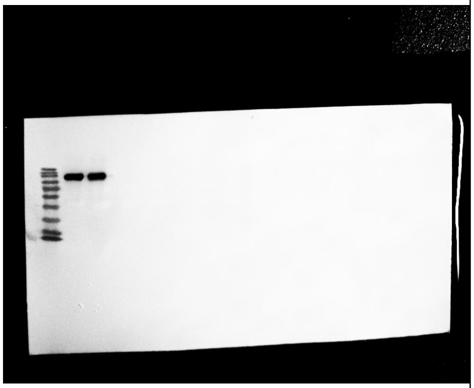		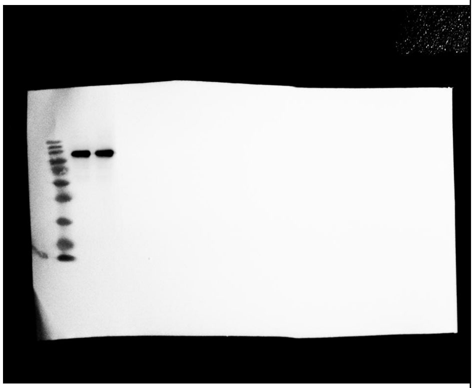	
FOXM1-SUCC	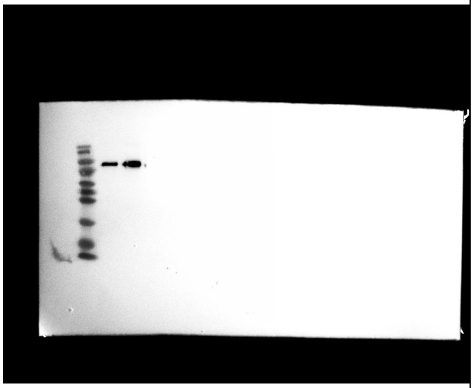		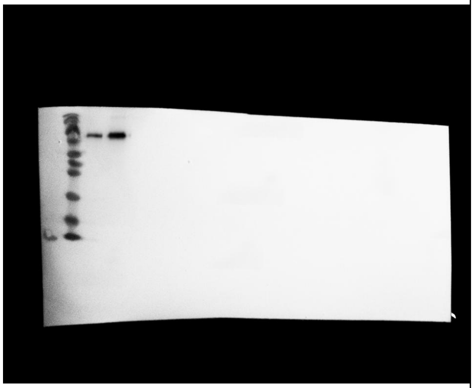	
GAPDH	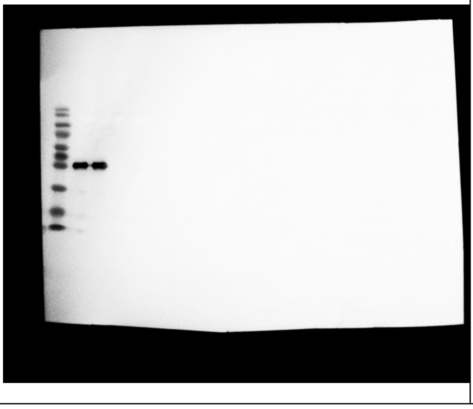		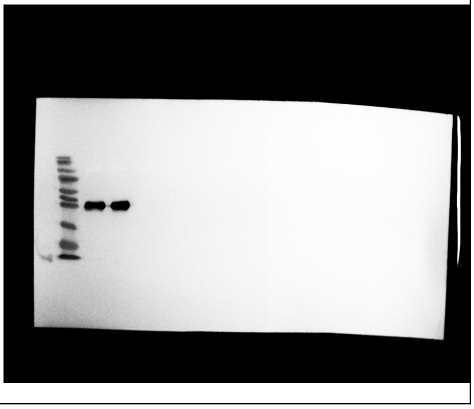	

Figure4F	vector	KAT2A	KAT3B	CPT1A	SIRT5	SIRT7
FOXM1-SUCC	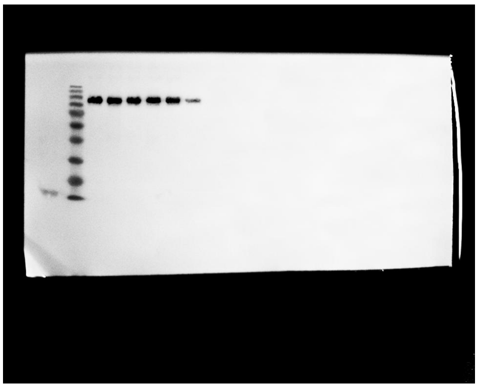					
GAPDH	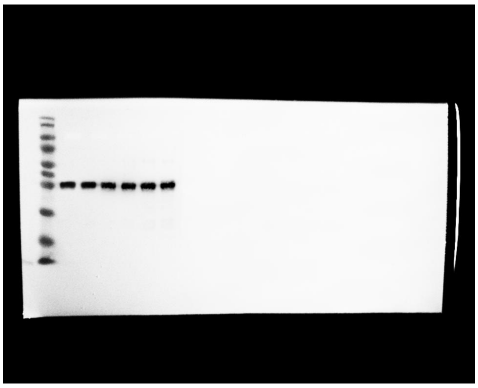					

Figure4 G+H	Input	IgG	SIRT7	Input	IgG	FOXM1
SIRT7	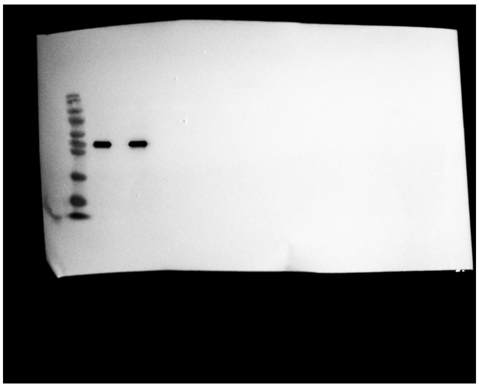			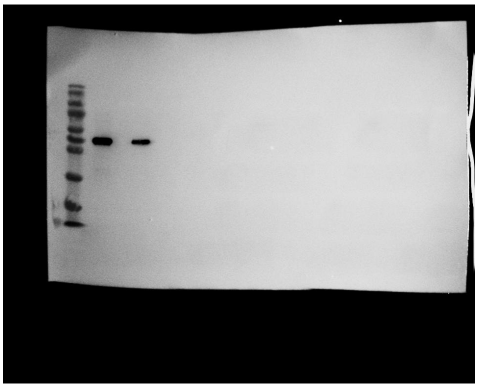		
FOXM1	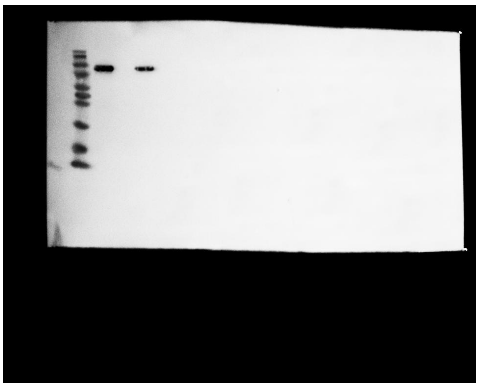			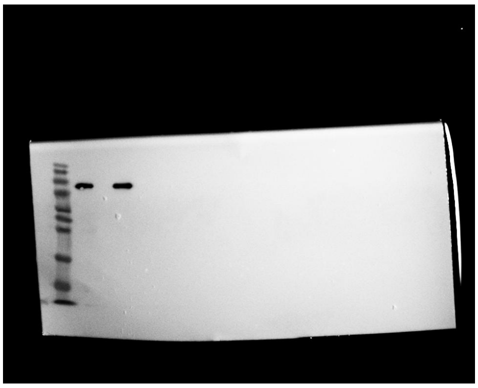		

<table>
<tr><td colspan="2" rowspan="2">Figure4I</td><td>vector</td><td>SIRT7</td><td>vector</td><td>SIRT7</td><td>vector</td><td>SIRT7</td><td>vector</td><td>SIRT7</td></tr>
<tr><td>Flag-FOXM1-WT</td><td>Flag-FOXM1-WT</td><td>Flag-FOXM1-K12S</td><td>Flag-FOXM1-K12S</td><td>Flag-FOXM1-K259S</td><td>Flag-FOXM1-K259S</td><td>Flag-FOXM1-K324S</td><td>Flag-FOXM1-K324S</td></tr>
<tr><td rowspan="2">IP: Flag</td><td>FOXM1-SUCC</td><td colspan="8">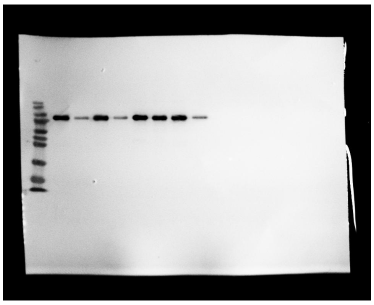</td></tr>
<tr><td>Flag-FOXM1</td><td colspan="8">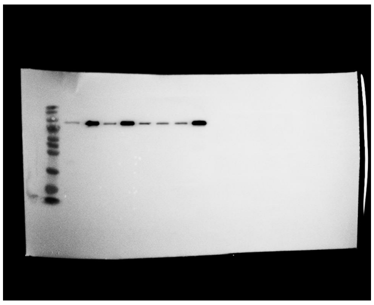</td></tr>
<tr><td rowspan="3">TCL</td><td>Flag-FOXM1</td><td colspan="8">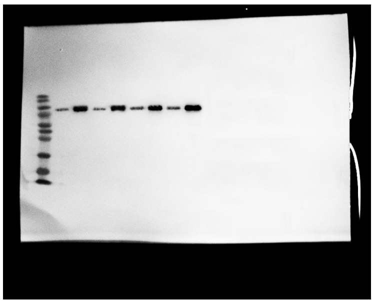</td></tr>
<tr><td>SIRT7</td><td colspan="8">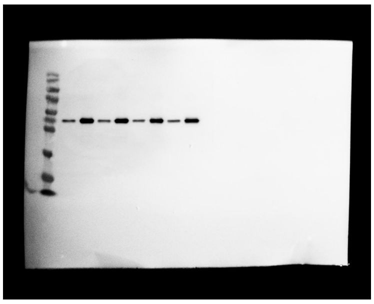</td></tr>
<tr><td>GAPDH</td><td colspan="8">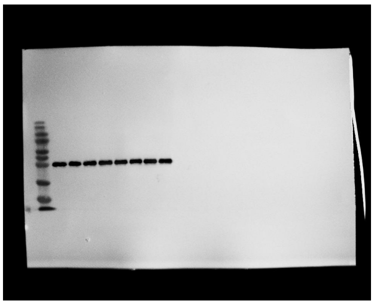</td></tr>
</table>

Figure5D	si-NC	si-SH3PXD2A-AS1	si-SH3PXD2A-AS1+vector	si-SH3PXD2A-AS1+SIRT7
FOXM1-SUCC	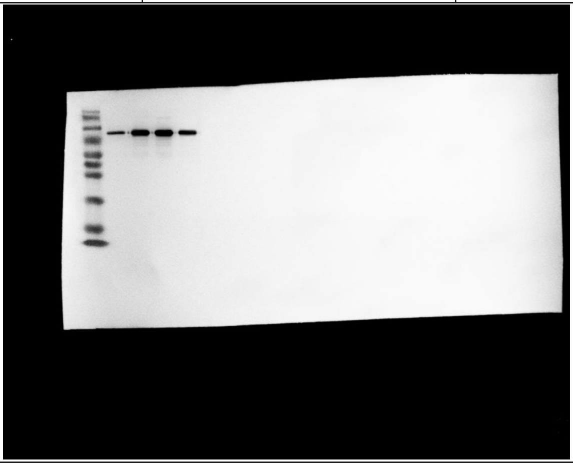			
FOXM1	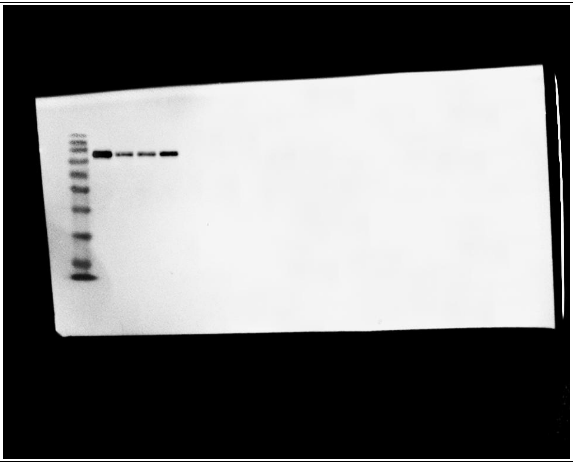			
GAPDH	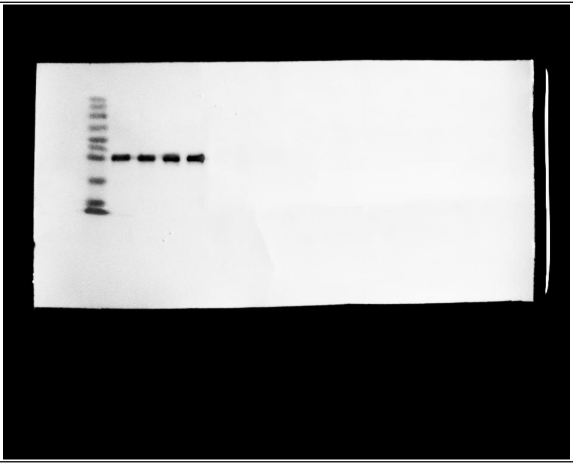			
